# Ontological Representation of Light Wave Camera Data to Support Vision-Based AmI

**DOI:** 10.3390/s120912126

**Published:** 2012-09-05

**Authors:** Miguel Ángel Serrano, Juan Gómez-Romero, Miguel Ángel Patricio, Jesús García, José Manuel Molina

**Affiliations:** Applied Artificial Intelligence Group, Universidad Carlos III de Madrid, Avd. de la Universidad Carlos III, 22, Colmenarejo, Spain; E-Mails: jgromero@inf.uc3m.es (J.G.-R.); miguelangel.patricio@uc3m.es (M.A.P.); jesus.garcia@uc3m.es (J.G.); molina@ia.uc3m.es (J.M.M.)

**Keywords:** visual sensor networks, light wave, structured light, time-of-flight, cognitive vision, ontology-based, ambient intelligence, social signal processing

## Abstract

Recent advances in technologies for capturing video data have opened a vast amount of new application areas in visual sensor networks. Among them, the incorporation of light wave cameras on Ambient Intelligence (AmI) environments provides more accurate tracking capabilities for activity recognition. Although the performance of tracking algorithms has quickly improved, symbolic models used to represent the resulting knowledge have not yet been adapted to smart environments. This lack of representation does not allow to take advantage of the semantic quality of the information provided by new sensors. This paper advocates for the introduction of a part-based representational level in cognitive-based systems in order to accurately represent the novel sensors' knowledge. The paper also reviews the theoretical and practical issues in part-whole relationships proposing a specific taxonomy for computer vision approaches. General part-based patterns for human body and transitive part-based representation and inference are incorporated to an ontology-based previous framework to enhance scene interpretation in the area of video-based AmI. The advantages and new features of the model are demonstrated in a Social Signal Processing (SSP) application for the elaboration of live market researches.

## 1. Introduction

AmI develops computational systems that apply Artificial Intelligence techniques to process information acquired from sensors embedded in the ambience in order to provide helpful services to users in daily activities. AmI objectives are: (i) to *recognize* the presence of individuals in the sensed scene; (ii) to *understand* their actions and estimate their intentions; (iii) to *act* in consequence.

The use of visual sensors in AmI applications has received little attention [1], even though they can obtain a large amount of interesting data. Some reasons are: the economic cost of visual sensor networks, the computational requirements of visual data processing, the difficulties to adapt to changing scenarios and the disadvantages with respect to other sensor technologies, such as legal and ethical issues.

In the last decade, new visual sensor technologies have updated the established concepts of the computer vision approaches. Time-of-Flight (ToF) technology provides both intensity and distance information for each pixel of the image, thus offering 3-dimensional imaging [2,3]. Structured light imaging allows to obtain an accurate depth surface for objects with an unprecedented resolution. Recently, the cost of these sensors has been dramatically reduced, which has lead to a widespread adoption of these technologies, now even present in consumer electronics like the Kinect™ peripheral for Microsoft XBox™ system.

New computer vision algorithms have been proposed to detect and track human movements from structured light and ToF sensors [4]. These works are mostly based on the definition of a model and motion of the human body. To name some application areas, ToF-based systems have been used in tracking algorithms for the detection of moving people [5], nose detection algorithms [6], body gesture recognition [7], hand tracking proposals [8,9], SSP to classify human postures [10] and Ambient Assisted Living to detect people falls [11].

Unfortunately, current approaches do not provide a well-defined model to represent the semantic details of the data, such as relationships or constraints, coming from new algorithms. The use of a conceptual model offers several advantages at a low cost. Formal models establish a common symbolic vocabulary to describe and communicate scene data while providing support for logic-based reasoning. Symbolic language is closer to human language, and therefore it is easy to interact and interpret system inputs and outputs. Reasoning, in turn, can be applied to check the consistency of the models and to infer additional knowledge from explicit information.

The formulation of models based on abstraction levels has led to the implementation of non-cohesive systems which are not able to fluently communicate among themselves. For this reason, it is necessary to provide new common and transverse knowledge layers among these levels including new semantic relationships. The goal of this strategy is the close interaction among semantically similar layers to the automatic generation of new knowledge. With the advent of new sensors, we advocate for the addition of a representation layer based on mereology and meronymy. Meronymy studies part-whole relations from a linguistics and cognitive science perspective. Mereology is a close concept, which concerns the formal ontological investigation of the part-whole relation and it is formally expressed in terms of first-order logic. The idea of employing a part-based layer to support the statements of the scene object abstraction level in a cognitive architecture has been previously suggested by Pinz *et al.* [12]. Our proposal goes further and seeks to provide a symbolic layer based on the formal definition, development patterns and implementation of part-whole relationships.

Symbolic data representations allow to develop cognitive models able to represent more accurately the complexity of the scene. These models can analyze systematically the knowledge of the scene to discover and describe data related with activities developed by a subject fusing its representation with high-level context knowledge—the set of circumstances surrounding a situation of interest that are potentially of relevance to its completion [13]. A key part of such analysis is currently supported by the approaches emerged from a cognitive view of the traditional computer vision techniques. The ties between meronymy and the current qualitative approaches [14,15] in cognitive vision—mainly focused on a qualitative description of spatio-temporal aspects [16]—must be regarded as crucial to narrow the gap of knowledge in activity recognition approaches.

This paper describes an ontology-based model for data acquired from recognition algorithms through light wave technology. This model is incorporated into a cognitivist [17] (According to Vernon's definition “Cognitivism asserts that cognition involves computations defined over symbolic representations, in a process whereby information about the world is abstracted by perception, represented using some appropriate symbol set, reasoned about, and then used to plan and act in the world.”) framework for contextual fusion of 2D visual information previously proposed by our research group [18–20]. The cornerstone of the framework is an ontological model designed according to the Joint Directors of Laboratories (JDL) fusion model [21] that represents sensor and context information stepped in several levels from low-level tracking data to high-level situation knowledge. The ontological model has been designed to promote extensibility and modularity. Each ontology level provides a skeleton that includes general concepts and relations to describe very general computer vision entities and relations. A general taxonomy of part-whole relationships for computer vision is proposed. The relationships are distributed along the levels of the model according to their abstraction. Several general pattern based on transitive part-whole relationships are proposed to cover the representation of the data to the level of accuracy currently achieved and to improve the quality of the inference process.

To illustrate the functioning of the extended framework a case study based on a SSP environment is presented. SSP aims at providing computers with the ability to sense and understand human social signals [22]. The example depicts a novel application of structured light cameras for live market researches. The goal is the formal representation of complex activity recognition and the automatic reasoning through ontologies. The example incrementally describes the activities representation through the presented model and the automatic structuring of event knowledge along the part-based level. Straightforward rules corresponding to a logic inference engine are attached to the example sections to demonstrate that the application is feasible.

The reminder of this article is organized as follows. Section 2 discusses theoretical issues in part-based representations. Section 3 includes an overall description of the new features added to our framework due to the use of novel sensors. Section 4 describes a symbolic layer which includes the proposal of a part-based taxonomy of properties for cognitive vision environments and a pattern which formalize the representation of those which are transitive. The pattern is depicted using the human body structure extracted from novel sensors. Section 5 details the configuration of event of interest for data extraction and propagation. The implementation issues are revisited in Section 6. Section 7 depicts a live market research scenario to detect interesting situations in the SSP area. Section 8 summarizes the conclusions obtained and proposes some directions for future work.

## 2. Theoretical Issues in Part-Based Representations

Meronymy has been subject of researches in linguistics, philosophy, and psychology.

From a philosophical point of view parts have been characterized as single, universal and transitive relations used to model, among others, the spatio-temporal domain [23]. This definition stay open since it was criticized by using an axiomatic representation which considers part-of a partial ordering relation [24]. Afterwards the representation was completed with the addition of new axioms [25].

Representations of part-based relations are founded on the Ground Mereology theory. The Ground Mereology establishes three principles [26]:

- Reflexive: Everything is part of itself.
  ∀*x*(*part_of*(*x, x*))
- Antisymmetric: Two distinct things cannot be part of each other.
  ∀*x, y*((*part_of*(*x, y*) ∧ *part_of*(*y, x*)) → *x* = *y*)
- Transitive: Any part of any part of a thing is itself part of that thing.
  ∀*x, y, z*((*part_of*(*x, y*) ∧ *part_of*(*y, z*)) → *part_of*(*x, z*))

These principles have been a source of discussions in meronymy due to the need to consider different kinds of part-whole relations and because some of them must be intransitive. Some examples can be found in [27].

The variety of semantic senses in part-whole relations drove researchers to look for a collection of part-whole relations. Winston *et al.* [28] developed a taxonomy founded on three linguistic and logical characteristics: functional, homeomerous and separable. These characteristics define a set of six meronymic relations: component-integral object, member-collection, portion-mass, stuff-object, feature-activity and place-area.

**Figure 1.**
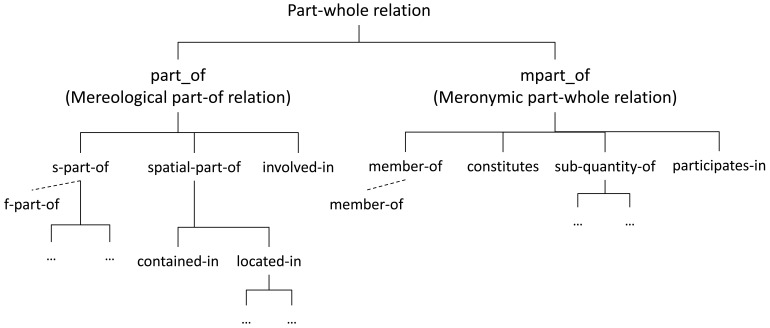
Keet *et al.*'s taxonomy of basic mereological and meronymic part-of relations.

Keet *et al.* [29] proposed a formal taxonomy of part-whole relations (see Figure 1) which implements a compromise solution for the “ontologically-motivated relations useful for conceptual modeling up to the minimum level of distinctions”. This taxonomy is particularly relevant since the properties are defined using categories of the DOLCE [30] upper ontology. The taxonomy by Keet *et al.* is extended in Section 4.1 to be applied in cognitive vision environments.

**Figure 2.**
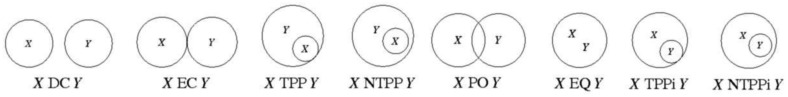
RCC-8 relations.

Interestingly enough, connectedness is a fundamental concept shared between the foundations of mereological and topological theories. As it is shown in mereotopological approaches [31], topology can be defined as a domain specific subtheory of mereology and mereology can be defined as a subtheory being topology primal. An example of the latter is the theory developed by Randell *et al.* [14], who propose the Region Connection Calculus (RCC). RCC defines the part-of relation in terms of the connection relation. RCC is an axiomatization of certain spatial concepts and relations in first order logic. The basic theory assumes just one primitive dyadic relation: C(x, y) read as x connects with y. Individuals (x, y) can be interpreted as denoting spatial regions. The relation C(x, y) is reflexive and symmetric. The subsets including Disconnected (DC), Externally Connected (EC), Partially Overlaps (PO), Equal (EQ), Tangential Proper Part (TPP), Non-Tangential Proper Part (NTPP), Tangential Proper Part Inverse (TPPi) and Non-Tangential Proper Part Inverse (NTPPi) (see Figure 2) have been proven to form a jointly exhaustive and pairwise disjoint set, which is known as RCC-8. Similar sets of one, two, three and five relations are known as RCC-1, RCC-2, RCC-3 and RCC-5.

Current capabilities in computer vision systems do not allow an easy recognition of mereological relationships from spatial inclusion assertions. Topological relationships between two entities, for example, TPP, NTPP, EQ or PO relations, are essential cues to detect part-whole patters; however, it is also necessary to detect a connection relation among the content and the container. On the other hand, we advocate for the combined use of spatial and mereological knowledge at different levels. A separate definition of theories can be used to classify and assert new knowledge. A clear example is the classification of subactivities. The spatial context of a subactivity can determine the relationship with the overall activity. Comparing products in the supermarket is part of shopping; however, comparing products can be part of cooking if the subject is in a kitchen. Sections 5.1 and 6.1 present a practical approach on the combination of topological and mereological relations and their implementation in our system.

## 3. Ontology-Based Computer Vision Model and Light Wave Technology Integration

The representation for new sensors data has been used in the framework for computer vision representation presented in [20]. This framework is based on an ontological model for the representation of context and scene entities. The ontological model is organized into several levels compliant with the Joint Directors of Laboratories (JDL) model for Information Fusion [21]. Each layer includes general concepts and properties to describe computer vision entities and relations at different abstraction level. Concepts that belong to a less abstract ontology are the building blocks of concepts corresponding to a more abstract ontology. Current implemented levels are:

- Tracking Entities level, to model input data coming from the tracking algorithms: track information (color, position, speed) and frames (to support the temporal consistency).
- Scene Objects level, to model real-world entities, properties, and relations: moving and static objects, topological relations, *etc.*
- Activities level, to model behavior descriptions: grouping, approaching, picking an object, and so forth.

The model has been designed to promote extensibility and modularity. This means that the general structure can be refined to apply this model to a specific domain. Local adaptations should not cause cascade changes in the rest of the structure.

Ontologies may contain both perceptual and context data. Perceptual data is automatically extracted by tracking algorithms, while the context data is external knowledge used to complete the comprehension of the scene. For example, the description of a sensorised static object—size, position, type of object, and so on—is regarded as context data.

Some changes are needed to model tracking data coming from novel devices. The priority to adapt these changes is to maintain the compatibility with the previous approach. The ontologies of the initial framework have been extended to include support for light wave data:

- An additional Euclidean dimension for the depth position of recognized objects. This is easily achieved by relying on the qualia approach [30] used in the original ontology model to represent properties and property values.
- A new definition of the concepts that represent human entities in the scene. Essentially, the current description of a subject in the scene, represented by the Person concept, is now associated with a description of anatomical joints and limbs. This description has been formalized according to existing patterns to represent part-whole relations with ontologies and current ToF-based computer vision models for articulated bodies.

The introduction of new devices requires upgrading the capacity of spatial representation in the model from two to three dimensions. These changes concern both perceptual data captured by light wave cameras and context data representing physical objects. The previous model followed the qualia approach used in the upper ontology DOLCE [30]. This modeling pattern distinguishes between properties themselves and the space in which they take values. The values of a quality—e.g., Position—are defined within a certain conceptual space—e.g., 2DPoint. To adapt the ontology-based model to this new quality space, the 3DPoint concept, which represents a position using three coordinates, is included as a subclass of PositionValueSpace, which represents the space of values of the physical positions.

Current Kinect™ algorithms are able to detect real-world entities; e.g., a person including data related to the human limbs and joints. Our ontology-based model represents these kinds of real-world data at the scene object level. However, these data also include low level information that should be represented as tracking entities to support the scene object assertions. Tracking entities level has been adapted to represent low level data of human members and joints—position, size, kinematic state, and so on—and this information is associated to the Person concept declared in the scene object level. The inclusion of limbs and joints is compliant to the previous version of the tracking entities ontology. The applied part-whole pattern (see Section 4.2) allows keeping backward compatibility. In fact, this model can combine 2D monocular cameras and light wave devices using the same set of ontologies.

## 4. Part-Based Symbolic Layer for Cognitive Vision Approaches

This section presents a part-based taxonomy of properties for cognitive vision environments based on some approaches discussed in Section 2. Afterwards a general ontology-based pattern to represent the transitive properties of the taxonomy is explained. To illustrate this pattern we have chosen the semantics of the human body and its parts. Thereby we fulfill the dual purpose of explaining the general pattern and its application to exploit the detection of human body structures using novel devices.

### 4.1. Part-Based Taxonomy of Properties for Cognitive Vision Environments

The identification of the underlying characteristics presented in Section 2 allows to discriminate between several kinds of part properties. The characteristics by Winston *et al.* are appropriate for cognitive vision representation because they are mainly supported by spatio-temporal foundations. However this set of characteristics is too small and do not allow a wide specialization of properties. Thus we have also taken into account the classification by Opdahl *et al.* [32] (see Table 1).

**Figure 3.**
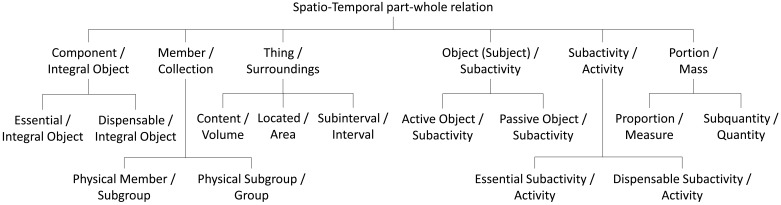
Proposed taxonomy of part properties for spatio-temporal aspects in vision-based systems.

The resulting classification is focused on properties which can be projected as spatial and temporal concepts captured by visual devices. Figure 3 shows the proposed taxonomy taking into account the spatio-temporal aspects in vision-based systems. We carry out an analysis based on characteristics of part properties. This analysis only considers the general characteristics of each property. We do not offer an exhaustive list of characteristics for each property because some of them do not characterize the property. Current classification can be reconsidered for a specific specialization according to a particular domain. It is considered that all the properties meet the Ground Mereology principles except transitivity.

Component/Integral object ( componentOf): This is a functional, separable, resultant and transitive property. The property is relevant for unidentified entities and scene objects. Thus it is mandatory to define a set of subactivities where the part can intervene. There are two subtypes: (i) Essential/Integral object ( essentialComponentOf) are those critical parts to identify a whole, for example, the chest of a body. Their characteristics, in addition to the inherited, are: mandatory, existential dependency and immutable; (ii) Dispensable/Integral object ( dispensableComponentOf) are those parts that are not crucial for recognition. Following the previous example, a hand can be regarded as a dispensable component for body recognition. Their corresponding characteristics are: optional and mutable.

Member/Collection ( memberOf): This property aims to redefine the identity of an entity through its assimilation to a group. The necessary characteristics of this property are separable, optional, mutability, shareability. Generally this property is intransitive when it is used for abstract sets of membership, for example, when a person is part of an organization. The subproperties are specialized in the spatio-temporal level where they can be detected according to proximity measures or similar kinematic features: (i) Physical member/Subgroup ( physicalMemberOf) which meets the mandatory characteristic because the parts only can be scene objects corresponding to context data or detected entities with physical features; (ii) Physical Subgroup/Group ( physicalSubGroupOf) which meets transitivity, homeomerousity and mandatory characteristics because parts only can be clusters of physical members.

Thing/Surroundings ( settledIn): This property defines a content relationship and an invariant connection between the part and the whole. It is only applicable between objects and entities with spatial or temporal representation. The general characteristics of this property are: homeomerousity, invariance, optional, immutability, shareability and intransitivity. The transitive, mandatory and existential dependency subproperties are: (i) Content/Volume ( containedIn) is exclusively used by spatial representations based on 3D points; (ii) Place/Area ( locatedIn) is exclusively used by spatial representations based on 2D points; (iii) Subinterval/Interval ( intervalOf) is used by temporal representations based on time intervals.

Object (Subject)/Subactivity ( involvedIn): This intransitive property defines the subjects that are involved in an activity. Its characteristics are functional, non-homeomerous, separable, optional and sharable. Objects and subjects with functional part properties in their definition are the main candidates to instantiate this property. The identified subproperties are not based on any characteristic but in our knowledge about the activity recognition: (i) Active Object/Subactivity ( activelyInvolvedIn) is instantiated when the object performs the activity; (ii) Passive Object/Subactivity ( passivelyInvolvedIn) is instantiated when the object is passively involved in the activity.

Subactivity/Activity ( participatesIn): Represents the relation among straightforward activities which participates in more complex activities. The main characteristic of this property are: functional, separable, homeomerous, transitive and sharable. The property can be divided in: (i) Essential Subactivity/Activity ( essentialSubActivityOf) if the subactivity is mandatory for the recognition of a more complex activity. Its specific characteristics are: mandatory, existential dependency and immutability; (ii) Dispensable Subactivity/Activity ( dispensableSubActivityOf) if the subactivity is not crucial to recognize a more complex activity. Its specific characteristics are: optional and mutability.

Portion/Mass ( portionOf): Necessary characteristics of this property are: homeomerousity, separability and intransitivity. Two transitive subproperties have been identified: (i) Proportion/Measure ( proportionOf) if the property is countable with a spatio-temporal measure. For example, a second is the sixtieth part of a minute. The corresponding characteristics are: functional, mandatory and existential dependency; (ii) Subquantity/Quantity ( quantityOf) if there does not exist a visual proportion between the part and the whole. For instance, the part of the water spilled from a cup. The inherent characteristic of this subtype is mandatory.

Stuff/Object ( madeOf): The constituent material can help to identify an object avoiding false positives in the entity detection process. This property is typically used in part-based taxonomies; however it can not be detected in the scope of vision systems.

Some other characteristics from Opdahl *et al.* classification have not been mentioned because they are already defined in the Winston *et al.* set of properties (e.g., abstraction and homeomerousity), have the same name but a different meaning (e.g., separability) or are not general (e.g., shareability). It is interesting to note that shareability can be seen as a cardinality restriction for specific cases of some relationships. For example, a chest only can be part of one body. These kind of situations become a problem if the relationship is transitive. In Section 4.2 we present a pattern to manage the semantic of these situations.

Some of the properties shown in the previous taxonomy are intransitive, for example, involvedIn and physicalMemberOf. Sometimes there are complementary transitive relations that can be used to propagate a property along another property. The corresponding properties of the previous examples would be participatesIn and physicalSubGroupOf. To illustrate this, let us suppose a person who is a physical member of a group and the same group is part of a bigger group. This procedure only requires to declare the physicalMemberOf property along the physicalSubGroupOf property to automatically assert that a person is a physical member of the bigger group. A wider and strongly related vision of this issue is the table developed in [33] which defines the conditions for the overall set of transitive interactions between different types of properties.

### 4.2. General Model for Ontology-Based Human Skeleton Representation

There are several existing ontologies designed to share and reason with structured data representing human anatomy [34]. Unfortunately, these ontologies have been developed in biomedical environments and define a complex conceptualization which is not useful to our needs. There are also other ontologies that represent the human body in a more simplified way [35]; however these ontologies are not designed to deal with sensor data in a cognitive environment. A general pattern based on part-whole relationships is proposed to cover the semantic representation of data captured using light wave sensors. The designed ontology adapts the patterns presented in [36] and follows the conceptualization of articulated bodies shown in [37] while keeping compatibility with DOLCE. Our proposal can be broadly adapted to other fields.

**Figure 4.**
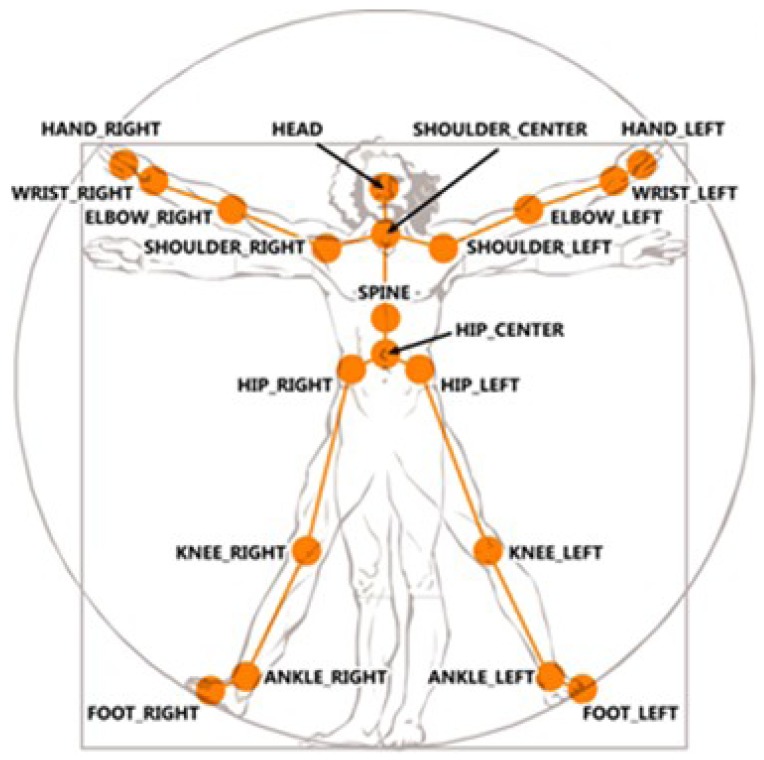
Joints captured by Kinect™ skeletal view.

Real-world knowledge achieves a more comprehensive representation organized through mereological relationships. A clear example is how the human mind divides the structure of a body in subjective parts. The current capabilities of Kinect™ skeletal view (see Figure 4 (reproduced from http://embodied.waag.org) allow the description of a detected person in terms of two kinds of attributes: (i) body members—hands, feet, thigh, and so forth; (ii) joints—shoulders, elbows, wrists, knees, and so forth. A conceptualization of the attributes detected and the limbs composed by these attributes is represented in the tracking entities level. Resulting concepts represent the parts of the human body which are embodied in the Person concept.

The properties named below ( partOf and partOf_ directly) correspond to the componentOf subtype of properties. The names have been modified to present the pattern in a general way since it can be applied to the rest of properties defined in Section 4.1.

Two properties are used to represent the part-whole relationships: (i) partOf; (ii) partOf_ directly—a partOf subproperty. partOf is a transitive property whose goal is establishing the correspondences between the parts and all the entities containing them. partOf_ directly defines the subjective relation among a part and the next direct level of composed entities. These properties are necessary since cardinality restrictions over transitive properties, such as partOf, are not allowed by OWL-DL. Therefore, partOf_ directly is used to define restrictions to maintain cardinality consistency, and partOf is used to infer both direct and indirect parts by means of transitivity and partOf_ directly property instances.

The previous ontology is extended with classes to represent direct parts—e.g., PersonPartDirectly—and the overall set of part-whole relationships—e.g., PersonPart. PersonPartDirectly subsumes direct parts of a Person such as Head, UpperLimb and LowerLimb. The classes hosting direct parts state existential range restrictions over partOf_ directly properties—e.g., partOf_ directly some Person. On the other hand PersonPart subsumes the set of parts of the Person concept. In this case, the direct parts of an UpperLimb concept, namely Arm, Forearm, Hand, Shoulder, Elbow and Wrist, are classified as subclasses of PersonPart; however they are not considered subclasses of PersonPartDirectly. The classes hosting direct and non-direct parts state existential range restrictions over partOf properties—e.g., partOf some Person.

To improve the consistency, cardinality restrictions—exactly 1—are stated over partOf_ directly as necessary conditions into the concepts corresponding to body members and joints. This means “a part only belongs directly to the next level entity and just to that entity”.

**Figure 5.**
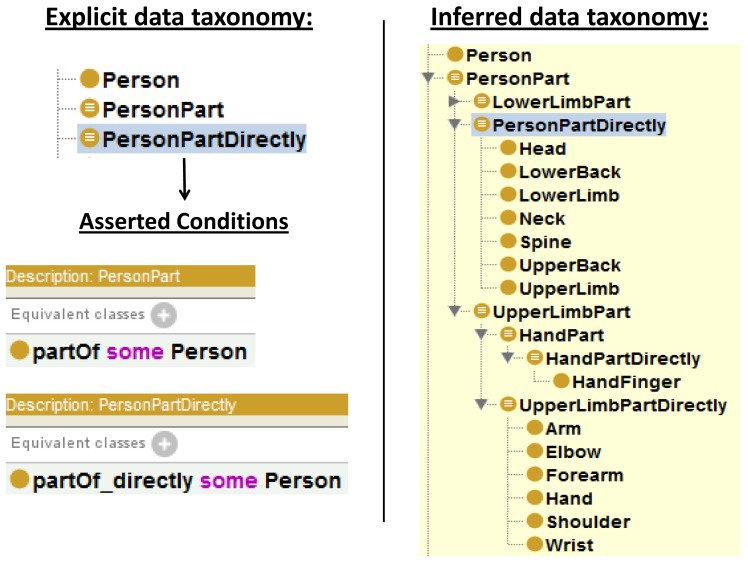
An example of explicit and inferred taxonomies.

The combined use of the part properties and the restricted classes leads reasoners to automatically infer new taxonomies derived based on part-whole relationships. Figure 5 illustrate an example of a taxonomy inferred from an explicitly stated taxonomy. Unfortunately, adding cardinality restrictions on each concept could significantly affect the performance of the reasoner. Some other configurations for this pattern are possible and also valid. This implementation tries to reduce the classification time while complying to the semantics of the human body domain.

**Figure 6.**
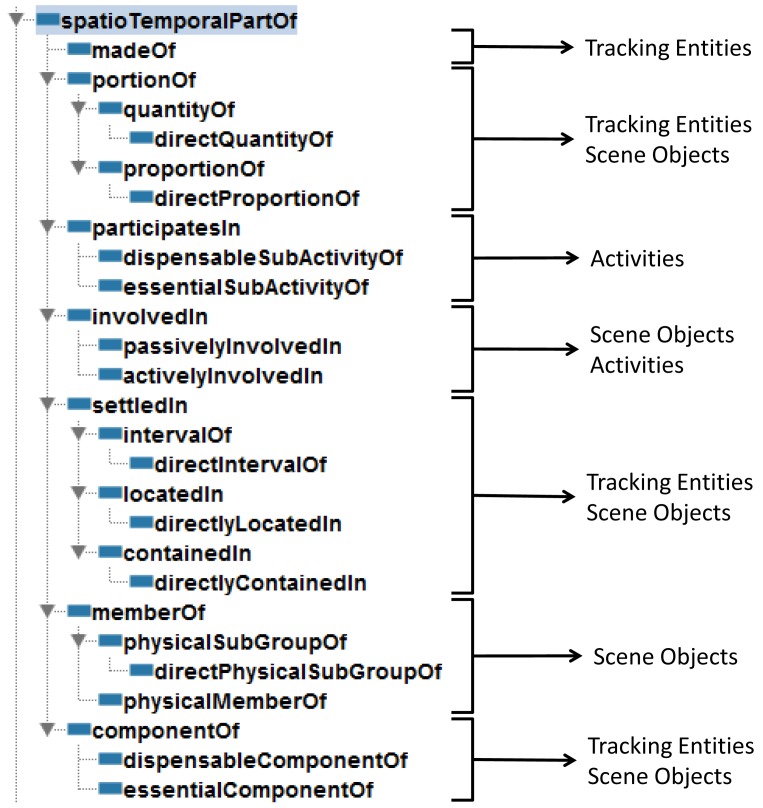
Spatio-temporal taxonomy with pattern representation.

Considering the combination of the taxonomy presented in Section 4.1 and the pattern above, we obtain a taxonomy to tackle with the spatio-temporal issues of a cognitive vision system. Figure 6 shows the implemented taxonomy, notice that some of the transitive properties do not include a direct property because it is implicit when the superproperty is transitive, for example, dispensableComponentOf and essentialComponentOf are regarded as direct properties because componentOf is transitive. Each subtaxonomy of properties is assigned to one or several levels forming a transverse layer through the model shown at the beginning of Section 3.

**Figure 7.**
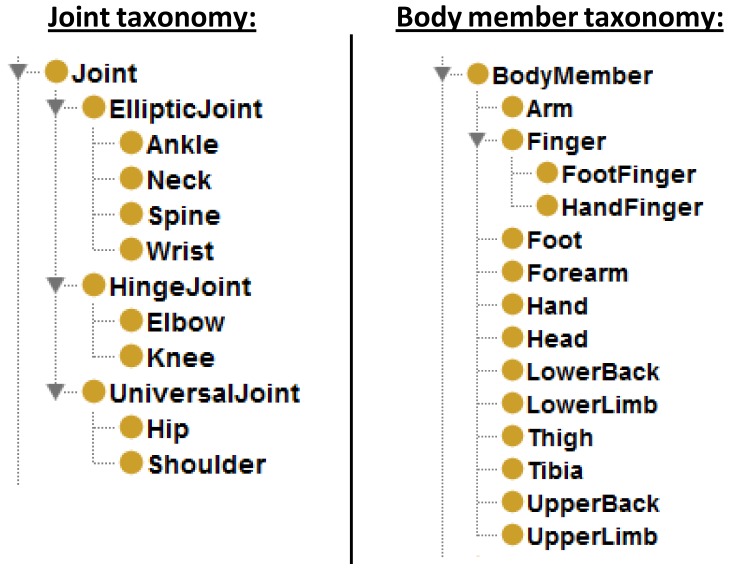
Explicit taxonomies for joints and body members.

The classification of joints is inspired by the virtual model shown in [37]. There are three types of joints (see Figure 7) depending on the degrees of freedom (DoF): (i) UniversalJoint, three DoF; (ii) HingeJoint, one DoF and two restricted DoF; (iii) EllipticJoint, three restricted DoF. Joint concepts store important data such as the articulated body members and the angle between them. These data is basic to maintain the consistency and to improve the semantic capacity of the model.

The model is designed by taking into account future changes in the granularity of the obtained data. New devices able to offer an accurate definition of the body members—e.g., the fingers of a hand—are easily adaptable. The larger the number of levels in the model, the greater amount of data is inferred. More details and additional information about data described in this section can be found in the authors' web page [42].

## 5. Part-Based Data Extraction and Propagation

There is an important amount of implicit knowledge surrounding the part-based approaches which should be extracted and used as a basis of the cognitivist models to improve the semantic richness and robustly justify the knowledge base reasoning.

### 5.1. Explicating Hidden Relationships Between Subclasses, Parts and Locations

The research by Winston *et al.* [28] shows the power to find implicit relationships using deductive reasoning based on syllogisms. The conclusion of this study indicates that there is a hierarchical ordering respectively between class inclusion, mereological inclusion and spatial inclusion which implies that “syllogisms are valid if and only if the conclusion expresses the lowest relation appearing in the premises”. Syllogism are a kind logical argument in which one proposition is inferred from two or more premises. A huge quantity of implicit relations can emerge from these inferences. The following example illustrates these assertions:

(1a) Peter is a physical member of a tourist group. (Mereological inclusion)
(1b) The tourist group is in the shop. (Spatial inclusion)
(1c) Peter is in the shop. (Spatial inclusion)

Ontologies have several advantages to carry out this kind of deductive reasoning because: (i) the hierarchical structure of ontologies is strongly related to the idea of class inclusion since terminological boxes represent concepts as general classes which host more specific or specialized classes; (ii) the mereological patterns to represent and reason with parts and the current reasoner's support for qualitative spatial approaches [38] provide the semantic support to apply this kind of arguments; (iii) the OWL 2 construct ObjectPropertyChain allows a property to be defined as the composition of several properties. Compositions enable to propagate a property (e.g., placedIn) along another property (e.g., partOf). The previously described syllogism is automatically handled by the following statement:

SubPropertyOf( ObjectPropertyChain(:partOf :placedIn) :placedIn)
  (Composition feature in OWL 2. http://www.w3.org/2007/OWL/wiki/New_Features_and_Rationale#F8:_Property_Chain_Inclusion Last accessed 12 April 2012)

Table 2 [39] shows the syllogisms' hierarchical ordering described through properties composition. Notice that the table's main diagonal compositions do not need to be declared since the properties are transitive.

### 5.2. Automatic Data Propagation of Events of Interest

Sometimes the knowledge originated in an entity component should be represented as knowledge directly attributable to the overall entity. A pattern for data propagation along the parts and to the whole can be deployed based on the pattern explained in Section 5. Another pattern from [36] is adapted to distribute the data concerning the events developed in the human body members. This pattern requires: (i) the creation of the hasEvent property, which indicates that a subject is the source of an event—these property can be also specialized to address more specific events; (ii) new classes—e.g. EventInBody or EventInUpperLimb—to classify events, which comprises all the events carried out by the body and their parts; (iii) the characterization of the partOf property as reflexive. As it is shown in Section 2, reflexivity is one of the principles of Ground Merology theory and dictates that “everything is part of itself”. These principles allows to include the whole entities in the taxonomy of parts. This causes the subsumption of the Person concept by the PersonPart class.

Classes which host instances of events state existential range restrictions over hasEvent properties, for example, EventInBody declares the restriction hasEvent someValuesFrom (someValuesFrom restriction. http://www.w3.org/TR/2004/REC-owl-features-20040210/#someValuesFrom Last accessed 08 May 2012) PersonPart and EventInUpperLimb states hasEvent *someValuesFrom* UpperLimbPart. To illustrate this, let us suppose the detection of an event in a hand. After the instantiation of the event and the corresponding property hasEvent, the reasoner propagates the event to the EventInBody and EventInUpperLimb classes. Thereby, events are classified by following an organization refined by anatomical levels. In addition, this pattern represents the affirmation “an event carried out by a person is an event executed by the person or any of its parts”.

**Figure 8.**
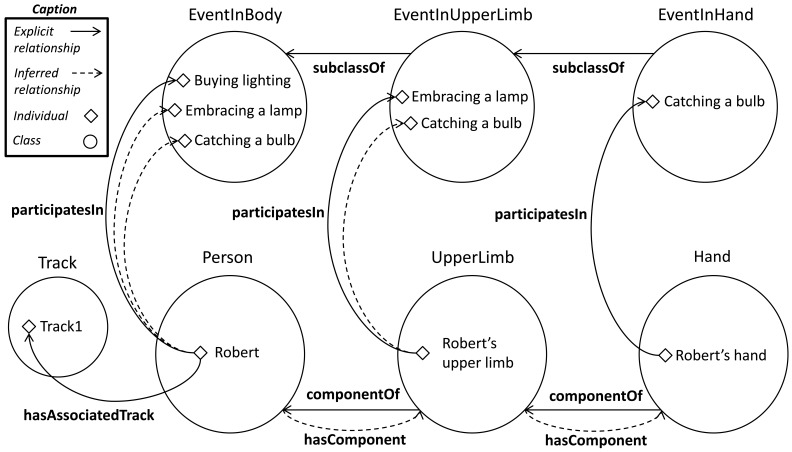
Inferred properties using composition between hasEvent and partOf.

This approach can be extended using a composition between the properties componentOf and participatesIn. Based on the relationship between an event and a body part, the relationships between parts of higher order that contains them and the event are automatically inferred. The following example syllogism and the Figure 8 depicts this extension:

(2a) Upper limb is component of Robert. (Explicit)
(2b) Robert's upper limb participates in embraces a lamp. (Explicit)
(2c) Robert participates in embraces a lamp. (Conclusion)

## 6. Implementation

The architecture presented in Section 3 has been implemented as a system prototype. The system has three basic inputs: a variable amount of a priori knowledge, sensor data coming from different information sources and data formalisms represented with ontologies. The ontologies include a set of terminological boxes (TBoxes), each of which containing sentences describing concept hierarchies. In turn, an assertional box (ABox) contains facts about individuals of the domain of discourse. These TBoxes make up the structure of the vision-based AmI symbolic representation. The ABoxes of these levels are filled with assertions from predefined context knowledge, previous inferences and sensor data.

The overall system is based on the RACER (Racer Systems GmbH & Co. KG. http://www.racer-systems.com/ Last accessed 05 April 2012) reasoner. The reasoner hosts the levels of the ontology-based computer vision model explained in Section 3; namely, tracking entities, scene object and activities [20]. RACER has been chosen because it includes support for different kinds of inference rules through the new Racer Query Language (nRQL), such as deductive, abductive, spatial and temporal [19].

Beyond the standard ontology reasoning mechanism based on subsumption, RACER also supports abductive and deductive rule-based inference. During the execution, abductive nRQL rules defined in a subontology create new instances that are asserted into the same level or into an upper level. Eventually, the creation of new instances as defined in the consequents of the rules draws instances corresponding to an interpretation of the scene in terms of the activity ontology. Deductive rules, in turn, are used to maintain the logical consistency of the scene. The consistency verifies whether all concepts in the TBox admit at least one individual in the corresponding ABox.

The output of the system is a coherent and readable interpretation of the scene logically justified from the low-level data to the high-level interpretation.

### 6.1. Spatio-Temporal Support

RACER is the first inference engine able to manage the spatial knowledge through an implementation of the RCC [14] (see Section 2 for definition) as an additional substrate layer. A substrate is a complementary representation layer associated to an ABox. The RCC substrate offers querying facilities, such as spatial queries and combined spatial and non-spatial queries. Although spatial instances from the ABox are not automatically connected with the RCC substrate, there is an identifying correspondence between them and the objects stored in the substrate.

**Figure 9.**
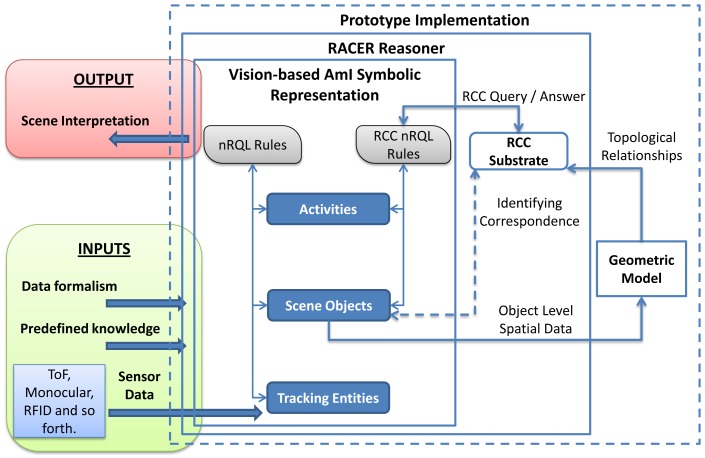
System implementation.

A significant amount of knowledge of scene objects and activity levels is obtained by abductive rules that include spatial properties in their antecedent. Figure 9 shows the integration of a geometric model in the system to dynamically calculate qualitative spatial relationships between scene objects. The geometric model receives spatial data from the scene object level. These data is instantiated into the Java Topology Suite (JTS) [43]. The JTS is an open source Java software library of two-dimensional spatial predicates and functions compliant to the Simple Features Specification SQL published by the Open GIS Consortium. JTS represents spatial objects in a Euclidean plane and obtains spatial relationships between two-dimensional objects quickly. Although OpenGIS spatial predicates and RCC-8 are not directly compatible, the output from the geometric model can be easily mapped from the OpenGIS format; in some cases, it only involves translating the name of the relationships. A correlation table between OpenGIS spatial predicates and RCC-8 can be found in [40].

Additional improvements could be implemented to increase the computation speed. It is interesting to highlight that checking object spatial relations, and particularly RCC relations, has a complexity O(n^2^) -the test must be performed between each pair of elements. Thus, it would be convenient to build a data structure able to maintain a hierarchical spatial partition on the Euclidean space. Currently, our framework does not support these improvements, which remains as a promising line for future work [41].

The temporal dimension can be represented as timestamps or time intervals. Timestamps are represented using snapshots of capturing data. Time intervals representation is directly supported by the RCC substrate thanks to their proper relationships [18]. The temporal dimension can be applied in both ways into the antecedent of rules.

## 7. Case Study: Live Market Research

Learning about relationships between the customer and the product at the point of sale is a very interesting knowledge in many economic fields, such as sales or marketing. Body gestures and spatial relationships contain useful knowledge about the sensations and intentions of shopping experiences. The model hereby presented can be used to automatically build live market researches based on the reactions and interactions of customers with the products.

Next subsections describe our system instantiation procedure and the expressiveness of the ontology model by presenting an activity recognition representation and a data propagation example. These subsections are depicted with rules to show its applicability in real environments.

### 7.1. Gesture Instantiation Procedure

A data set containing the skeleton representation of 11 people was designed to test the new representation. These body structures were captured by using a Kinect™ sensor. For each person five types of upper limbs gestures were stored: down, open, up, diagonal and akimbo. A control system based on the OWL API [44] functionalities automates the assertion of data in the form of axioms from the capture device to the ontology formalism. The control system manages the classification of the individuals received from the Kinect™ sensor, the explicit property instantiations such as partOf_ directly and the instantiation of properties that represent the articulation of body member through a joint. The control system also manages the automatic calculation of data values from the received data, such as the size of the body members and angles formed between them.

**Figure 10.**
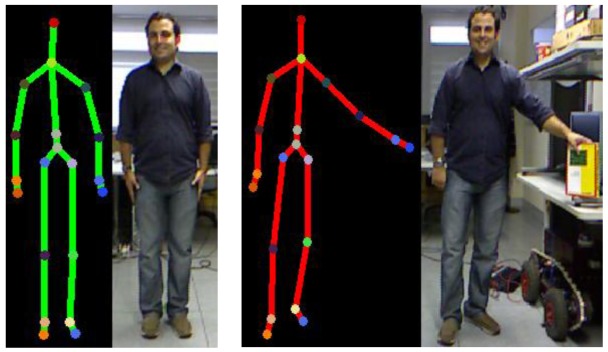
Gesture instantiation and action example.

An data instantiation example to describe a left upper limb with down gesture for the person in Figure 10 would include: (i) classification of joint instances (see Figure 7); (ii) partOf_ directly property instantiations (see Figure 5); (iii) joint positioning data.

### 7.2. Activity Recognition Example: Touching a Product

Activity recognition usually requires composition of simple activities along the time. Therefore temporal analysis is required in order to recognize complex activities [7]. Our ontology model is expressive enough to represent the temporal dimension of the activities. The representation capabilities resulting from the combined use of Kinect™ and the ontology-based model offer simple but very expressive tools to detect interesting activities for a market research confection.

Relevant activities for current market researches may be: stand in front of, look at, point at and touch a product. Recognition of simple interactions between different body members and objects regarded as context data can be detected finding the spatial relationship between these elements. The process becomes more robust if the object includes sensors (e.g., RFID and accelerometer) able to provide different kinds of features—id, location and kinematic state.

**Figure 11.**
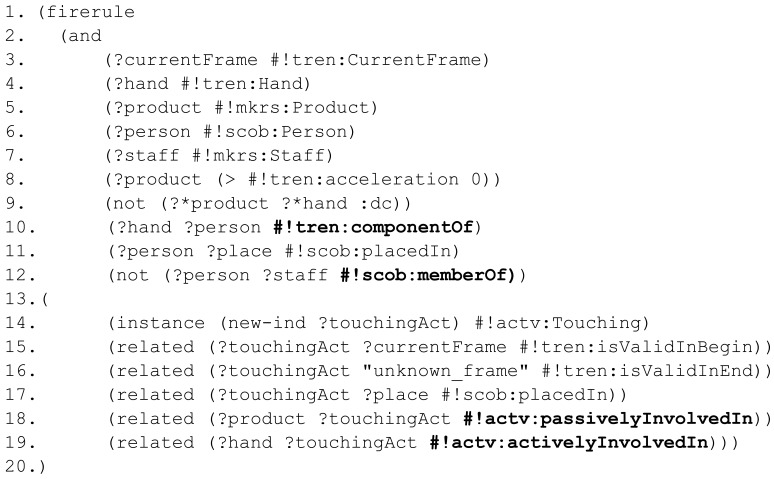
Rule to exemplify expressiveness.

In order to demonstrate the expressiveness of our representation, a syntactically relaxed nRQL—the query language of the RACER reasoner—rule is presented in Figure 11. The variables of the rule are denoted with a question mark at the beginning of their names (?), variables belonging to the RCC substrate are labeled adding a star (?*), concept types start with a hash (#) and RCC-8 relationships are labeled with a colon (:). To the existing namespaces, tracking entities (#!tren:), scene objects (#!scob:) and activities (#!actv:), a new one is added to group all the specific information related to market researches (#!mkrs:). The syntax of nRQL has been slightly simplified to make them more readable. The following rule detects touching activities between people and sensorized objects.

First, different variables that act along the rule are declared (3–7). The rule checks if the object involved in the situation is currently moving (8). This statement can also be used as a trigger of the rule. Afterwards, the rule checks if there is a spatial relationships between the moving Product and a Hand (9). The place of the person is assessed in (10–11). Finally, to discriminate between clients and employees, the rule considers if the person involved in the action is member of the staff (12). Identifying capability is referred in future work. If the antecedent conditions are satisfied, the consequent is applied. The consequent creates a Touching activity (14) with a known beginning (15) and an unknown ending (16). The spatial location of the activity is bounded by the location of the person who performs the activity (17). passivelyInvolvedIn and activelyInvolvedIn relationships among the new activity with the passive object (18) and the active subject (19) are also stated in the consequent. The resulting activity has been defined according to spatio-temporal criteria and part-based relationships.

**Figure 12.**
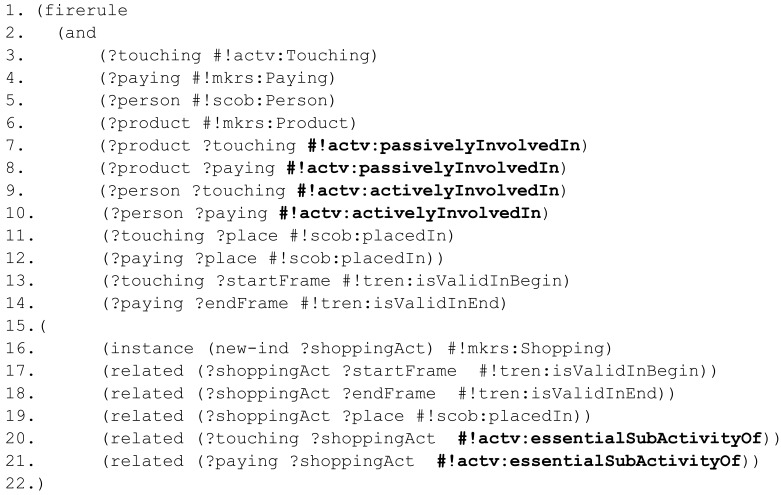
Simplified rule to recognize shopping.

The Touching activity is candidate to be classified as a subactivity of Shopping. To recognize the Shopping activity it is required to recognize a sequence of subactivities (e.g., touching the product, trying the product, interacting with the staff, paying for the product) where the same active subjects and passive objects are involved in the same place and time. For the sake of simplicity a rule which only recognizes the spatial dimension of a Touching and a Paying activity is showed in Figure 12.

At the beginning of the antecedent a set of variables are declared (3–6). Then, the same objects, subjects and places are identified in the subactivities (7–12). Finally, the starting and ending timestamps of the activities sequence are retrieved (13–14). The consequent creates a Shopping activity whose validity time interval is bounded by the starting point of the former activity and the ending point of the latter activity (16–18). The coincident place of the subactivities and the mereological properties between the subactivities and the overall activity are eventually asserted (19–21).

Crucial data is inferred from the former to the latter rule. Thanks to the interaction between the mereological and the geolocalized layers, the rules acquire more flexibility and the amount of relationships between concepts grows, which improves the completeness of the model. Imagine that the subactivities are detected in different places.

- touchingAct placedIn GroundFloor
- payingAct placedIn FirstFloor

The system can store mereological data stated to describe invariant context relationships such as:

- GroundFloor containedIn Shop
- FirstFloor containedIn Shop

In both cases, using the compositions described in Table 2, new relationships are inferred.

- touchingAct placedIn Shop
- payingAct placedIn Shop

**Figure 13.**
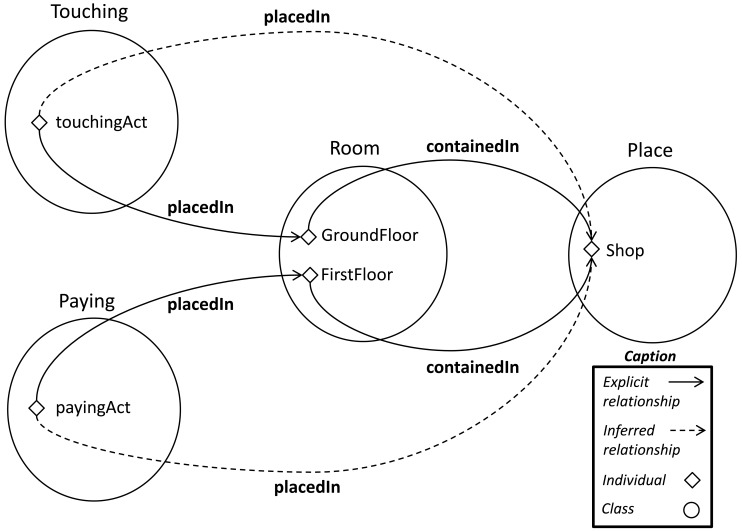
Representation of the inferred placedIn relationships.

Even though the activities have been detected in different places, the latter rule is fired because there is a common location for both activities (see Figure 13). Following the reasoning, an appropriate spatial environment ( Shop) is allocated to the overall activity (19).

### 7.3. Data Propagation Example: Touching a Product

Many data relationships are automatically propagated from the consequent's assertions of the previous section. In the first rule (19) of the previous section, a Hand is declared as active subject of the Touching subactivity. However, in the latter rule (9–10) a previously unstated assertion includes a Person as active subject of this subactivity. The pattern explained in 6.2 justifies the propagation of activity relationships for all the parts which contains the part performing the activity. When the Hand was declared as an active subject, the objects containing it were also inferred as active subjects.

- upperlimb activelyInvolvedIn touchingAct
- person activelyInvolvedIn touchingAct

Data propagation enable to choose the level of granularity of the information retrieval tasks and to assess data from multiple perspectives. The following query would retrieve the interactions among the people and the upper limbs, and the products during a campaign (it is assumed that, during a campaign, the products are located in the same place).

**Figure 14.**
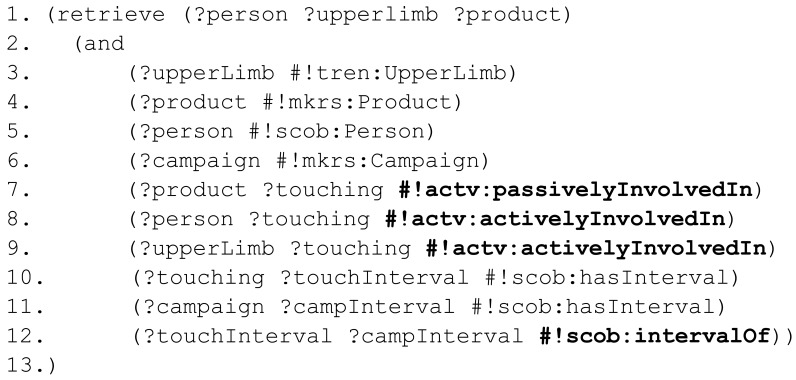
Query for different interactions during a campaign.

The query in Figure 14 retrieves different levels of active subjects ( Person and UpperLimb) of Touching activities for all the products on sale (1). Then query variables are declared (3–6). The Product, Person and UpperLimb of the same Touching activities are retrieved (7–9). From these set of activities, only those whose validity time interval is within the validity time interval of the campaign (10–12) are chosen.

The extracted information is helpful for answering abstract questions such as: “What is the visibility of this product?” A very rough answer would be the number of people who have interact with it. The level of doubts involved in the purchase decision can be also measured if we count the number of interactions of each user with the product. An extended model able to distinguish between right and left limbs could be used to assess the quality of the product accessibility.

**Figure 15.**
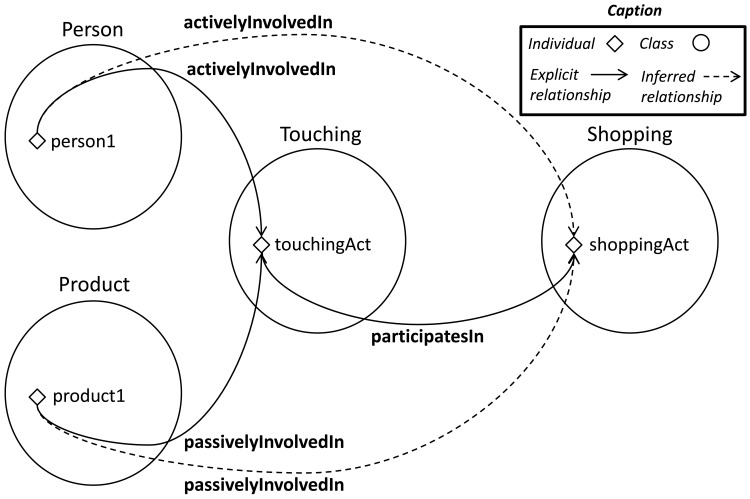
Representation of the inferred involvedIn relationships.

Another example of propagation is the automatic assignment of subjects and objects in composed activities. The first rule of the previous section states a Person and a Product as the active subject and passive object of a Touching subactivity. The system automatically connects these individuals as active subject and passive object of the shoppingAct individual when the touchingAct subactivity is detected participating in a Shopping activity individual (see Figure 15). This process is repeated, thanks to the composition explained in Section 4.1, each time a participatesIn property is instantiated.

## 8. Conclusions and Future Work

This paper proposes an update of the cognitivist models towards part-based representations. To do so, the work presents a theoretical taxonomy of mereological relations from a computer vision perspective. Using the Component/Integral object relationship of the taxonomy, we developed a general ontology-based model for formal representation of the human body semantics using part-whole patterns and data propagation patterns. The model has been embedded into a previous computer vision framework by relying on part-whole patterns and DOLCE recommendations. The proposal includes Kinect™ skeletal view data representation with backward compatibility. To illustrate the functioning of the extended framework, a case study for live market research has been described by presenting a data instantiation procedure and some examples of activity recognition representation and data propagation. These examples are able to represent semantically complex relationships through the interpretation of the user interactions with the context. The main advantages of this model are the general representation for further domain extensions and the logical capabilities for automatic inference of high-level relationships. Both advantages provide support for more sophisticated activity analysis.

Future research will be based on specific knowledge about the features of the users of a service. An important feature is the identity of a subject, which allows the differentiation among individuals. Kinect Skeletal View™ provides very significant data to recognize individuals, such as the shoulder width, the head width, the body height, the length of the limbs, and so forth. Market research data will be organized through automatic recognition of the gender and the age of the study subjects. We sense that Kinect Skeletal View™ can provide the ability to distinguish at least age ranges, such as child, adult or elder. Knowing the nature of the data, the research may be probably addressed towards fuzzy sets.

In addition, future works will address the completion of a full market research and the application of the entire model to a real life scenario combining monocular and light wave sensors. This application should include a probabilistic mechanism to reason with real world data asserted in the model, which may be imprecise or uncertain.

## Figures and Tables

**Table 1.** Set of characteristics to classify part-whole relations.

**Characteristic**	**Definition**
**Functional**	Parts are in a specific spatial/temporal position with respect to each other supporting their functional role with respect to the whole.
**Homeomerous**	Parts are visually similar to each other and to the whole to which they belong. Parts and aggregates belong to the same class.
**Separable**	Parts can be physically disconnected from the whole to which they are connected and can be detected without being part of a particular aggregate object. The opposite characteristic is **Invariance**.
**Resultant**	A part provides at least one property that extends to the whole.
**Mandatory**	An object of a particular class must be detected to declare the existence of an aggregate object. The opposite characteristic is **Optional**.
**Existential dependency**	A single and always the same occurrence of an object is critical for the life of the aggregate.
**Mutability**	A particular part object can be replaced in the aggregate object by another equivalent part without losing its identity. The opposite characteristic is **Immutability**.
**Shareability**	An object can be part of more than one aggregate object at the same time.
**Transitivity**	An object A is part of an aggregate B, the aggregate B is in turn part of another aggregate C, then A is also part of C. The opposite characteristic is **Intransitivity**.

**Table 2.** Composition of properties.

**⊗**	**hasClass**	**partOf**	**placedIn**
**hasClass**	hasClass	partOf	placedIn
**partOf**	partOf	partOf	placedIn
**placedIn**	placedIn	placedIn	placedIn
